# Thiol-Affinity Immobilization of Casein-Coated Silver Nanoparticles on Polymeric Membranes for Biofouling Control

**DOI:** 10.3390/polym11122057

**Published:** 2019-12-11

**Authors:** Xiaobo Dong, Halle D. Shannon, Atena Amirsoleimani, Gail M. Brion, Isabel C. Escobar

**Affiliations:** 1 Department of Chemical and Materials Engineering, University of Kentucky, Lexington, KY 40506, USA; xiaobo.dong@uky.edu (X.D.); halledanielle218@uky.edu (H.D.S.); 2 Department of Civil Engineering, University of Kentucky, Lexington, KY 40506, USAgail.brion@uky.edu (G.M.B.)

**Keywords:** silver nanoparticles (AgNPs), biofouling, leaching, ultrafiltration, *Serratia marcescens*

## Abstract

Silver nanoparticles (AgNPs) have been widely studied for the control of biofouling on polymeric membranes due to their antimicrobial properties. However, nanoparticle leaching has posed a significant impediment against their widespread use. In this study, a one-step method of chemically embedding AgNPs on cellulose acetate (CA) membranes via their affinity to thiol group chemistry was investigated. The operational efficiency of the membranes was then determined via filtration and biofouling experiments. During filtration study, the average flux values of pure CA membranes was determined to be 11 ± 2 L/(m2·hr) (LMH), while membranes embedded with AgNPs showed significant increases in flux to 18 ± 2 LMH and 25 ± 9 LMH, with increasing amounts of AgNPs added, which is likely due to the NPs acting as pore formers. Leaching studies, performed both in dead-end and crossflow filtration, showed approximately 0.16 mg/L leaching of AgNPs after the first day of filtration, but afterwards the remaining chemically-attached AgNPs did not leach. Over 97% of AgNPs remained on the membranes after seven days of crossflow leaching filtration studies. *Serratia marcescens* were then used as target microorganisms in biofouling studies. It was observed that membranes embedded with AgNPs effectively suppressed the growth of *Serratia marcescens*, and specifically, membranes with AgNPs displayed a decrease in microbial growth by 59% and 99% as the amount of AgNP increased.

## 1. Introduction

Membrane fouling is a major issue affecting polymeric membranes used for water treatment applications [1], and is due to the accumulation of colloids, biological matter, organic matter and scale formation upon the membrane [1,2]. Among these, biofouling, due to the accumulation of microorganisms on the membrane, plays a significant role by affecting the useful life of polymeric membranes [3,4]. Specifically, biofouling consists of several steps, being the deposition of bacteria on the membrane, the growth of bacteria into colonies, and the emission of extracellular polysaccharides (EPS) and other organic matter that lead to the formation of biofilms on the membrane [4,5]. Due to the difficulty in removing biofilms [3,6], a large field of research is about developing and/or improving the anti-biofouling properties of polymeric membranes. 

Silver nanoparticles (AgNPs) have been widely studied for their excellent antimicrobial performance [7,8,9,10]. The mechanism of AgNPs’ antimicrobial properties is still not fully understood, but these properties thought to arise from direct and indirect contact [11,12]. When AgNPs directly contact bacteria, the particles can adhere onto the surface of cells via electrostatic interactions, or Van der Waals forces, then penetrate inside the cells. 

After AgNPs penetrate into the cells, they can generate reactive oxygen species (ROS) and free radicals, which induce cellular toxicity and oxidative stress, leading to irreversible cell damage. Also, inside the cells, the signal pathways of bacteria can be modulated by AgNPs [12,13,14]. When AgNPs do not directly contact bacteria, they can interact with bacteria via releasing silver ions (Ag^+^). Then the ions can penetrate into the bacterial cell. Inside the cell, Ag^+^ can interact with the sulfhydryl groups of enzymes and proteins to deactivate enzymes and proteins in the bacteria [15]. Moreover, it is reported that Ag^+^ can form complexes with nucleic acids that prevent the division and reproduction of cells [11]. 

Membrane biofouling is characterized by the uncontrolled growth of microorganisms on membranes that leads to declines in efficiency and membrane life. AgNPs have been widely investigated in membrane fabrication as biofouling control agents. Several approaches of adding the nanoparticles to membranes have been investigated; for example, direct blending of AgNPs into dope solutions and followed by phase inversion [16,17,18], surface modification of polymeric membranes with the addition of AgNPs [19,20,21], layer-by-layer (LBL) assembly depositing AgNPs to form nanofiltration and reverse osmosis membranes [22,23], among others. However, when added to membranes, AgNPs have been observed to leach [12,24,25]; thus, incorporating them on membranes to provide antimicrobial properties, while minimizing leaching, is a challenge and the focus of this study.

The target bacteria for this study was *Serratia marcescens* (ATCC 13880), which is a gram-negative, facultative anaerobic bacterium [26] that can grow under conditions of 5–40 °C and pH ranges of 5–9 [27]. It is widely existent in nature water, soil, plants, rodents and hospitals [26]. As an opportunistic pathogen, it has been known to cause urinary tract, ocular lens, wound and respiratory tract infections through contact [26,28], and it has been reported to be resistant to antibiotics [28]. Some biogroups of *S. marcescens* can produce red pigments called prodigiosins in the environment when sufficient molecular oxygen is available, which makes microbial growth easily identifiable. Studies have shown that the non-pigmented strains of *S. marcescens* are often more resistant to antibiotics than the strains that can produce pigments [26]. 

Previously, a method to chemically attach casein-coated AgNPs to membranes modified with thiol groups was reported, obtaining successful results of simultaneously preventing biofouling on the membranes and minimizing leaching [24]. However, this method involved several post-synthesis modification steps that could impede the scale up. In this study, a one-step method of chemically incorporating AgNPs onto membranes was investigated. Specifically, AgNPs were chemically incorporated into a polymer complex, which was then blended with cellulose acetate (CA) to prepare the dope solution. Membranes incorporated with AgNPs were, thus, obtained without any post-synthesis modification procedures. The stability of AgNPs in the membrane matrix was determined, and the anti-microbial performance of the polymeric membranes incorporated with AgNPs was investigated.

Previous work that used thiol groups to immobilize AgNPs on membranes did this through a series of post-synthesis modification steps [24]. However, work on the membrane scale up using slot die casting has shown that ideally, membranes should be cast in one-step processes [29]. Therefore, the novelty here was to immobilize AgNPs on CA membranes using a one-step process. Specifically, the AgNPs were chemically blended with GMA and CYS prior to preparing the CA dope solution, and then blended into the dope to cast the membranes. Furthermore, the use of digitized colors to characterize microbial growth on membranes presented here is novel. 

## 2. Materials and Methods

### 2.1. Materials 

Cellulose acetate (CA, average *M*n ~30,000) was purchased from Sigma Aldrich (St. Louis, MO, USA). Glycidyl methacrylate (GMA, liquid, 97% stabilized with 100 ppm 4-methoxyphenol) was purchased from Alfa Aesar (Haverhill, MA, USA). The solvent used to dissolve polymers in this study was n-methyl-2-pyrrolidinone (NMP), purchased from Sigma Millipore (Burlington, MA, USA).

In the polymerization reaction, toluene (HPLC UV-grade) was purchased from Pharmo-AAPER (Shelbyville, KY, USA) and benzoyl peroxide (97% dry wt, wet with 2.5% water) was purchased from VWR International (Radnor, PA, USA). 2-aminoethanethiol (cysteamine, CYS) was purchased from TCI (Portland, OR, USA) and dimethyl sulfoxide (DMSO) was purchased from VWR International (Radnor, PA, USA) in order to functionalize the epoxide groups of the polyGMA. Bovine Serum Albumin (BSA) was purchased from VWR Life Science (Radnor, PA, USA).

The AgNPs (70.37% *w*/*w* Ag^0^) incorporated into the membranes were casein-coated and manufactured by Laboratorios Argenol (Zaragoza, Spain) via the irradiation technique and stabilized using casein. They were provided by Professor Dr. Vinka Craver from the University of Rhode Island (URI, Kingston, RI, USA). 

The nutrient agar solution was made from Difco^TM^ Nutrient broth, agar powder and sodium chloride (NaCl, 99.5%), which were all purchased from VWR International (Radnor, PA, USA). Bacterial strain, #13880 *Serratia marcescens* subsp. *marcescens* Bizio was purchased from ATCC (Manassas, VA, USA). Acetone was purchased from VWR International (Radnor, PA, USA) and ethanol was purchased from Sigma Millipore (Burlington, MA, USA). 

### 2.2. Reactions Preparing the Polymer Complex

#### 2.2.1. Polymerization of GMA

First, the polymerization of mono-GMA took place in an Erlenmeyer flask according to the previously developed method [24,30,31]. Two syringe needles were inserted into the flask so that nitrogen could enter the flask through one and leave through the other, creating a nitrogen atmosphere used to prevent undesirable reactions with oxygen in the atmosphere from occurring [24,30,31]. In the Erlenmeyer flask, 30 mL mono-GMA was mixed with 70 mL toluene, 0.2 g benzoyl peroxide by a magnetic stirring bar. Toluene was used as the solvent and reaction medium, and benzoyl peroxide acted as the initiator of the polymerization reaction [24,30,31]. The reaction was held at a constant temperature of 65–70 °C and stirred constantly for 6 h [24]. The polymer was then collected, dried and broken into small pieces. 

#### 2.2.2. Preparing polyGMA-CYS-Ag Polymer Complex

Theoretically, the reaction of polyGMA and cysteamine (CYS) is a 1:1 reaction with the primary amine of the CYS attaching to the GMA epoxide group; however, it has been reported that the CYS-GMA reaction has an efficiency of 58% [32]. Therefore, 1 g (0.007 moles) of polyGMA and 0.935 g (0.012 moles) of CYS were needed for the reaction. 1 g of polyGMA was combined with 0.935 g CYS, 5 mL DMSO, 5 mL deionized water (DI water) and a magnetic stirring bar in a 50 mL beaker. The amount of DMSO and DI water were based on previous studies [24,30]. This mixture was covered with Parafilm and stirred at 600 rpm, 60 °C for 6 h. The solvents and experimental conditions were based on literature [24,32]. The contents of the beaker were centrifuged for 3 min at 112× g (times gravity) to harvest the polyGMA-CYS from the solution. The collected polyGMA-CYS was then rinsed with DI water three times. 

**Figure 1:**
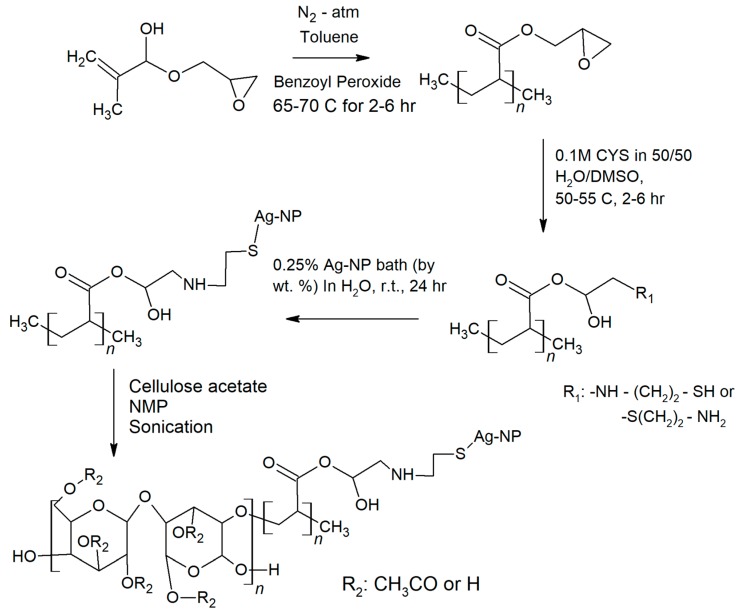
Cellulose acetate/silver nanoparticle (CA/AgNP) immobilization reactions: 1. Polymer-ization of monoGMA; 2. Attachment of CYS on polyGMA; 3. Attachment of casein-coated AgNP on the thiol group; 4. Blend of CA and polyGMA-CYS-AgNP.

The reaction of AgNPs to CYS was accomplished via the thiol group of CYS, which is a reaction with 1:1 atomic ratio. This reaction, shown in Figure 1, was carried out using casein-coated AgNP (70.37% *w*/*w* Ag^0^). The polyGMA-CYS was reacted with 1.08 g (0.007 moles) casein-coated AgNP, 10 mL DI water, and a magnetic stirring bar in a 50 mL beaker for 24 h, with stirring continuously at 600 rpm. The remaining solution was collected and centrifuged for three min at 112× g to collect the polyGMA-CYS-AgNP from the solution. The solution was then rinsed with DI water in the centrifuge at 12 × g for 3 min and repeated three times. The polymer complex was then freeze-dried for 12 h and the collected polyGMA-CYS-AgNP polymer complex powders were cryo-milled twice after being dried.

### 2.3. Membrane Fabrication 

Five dope solutions were prepared for casting. 18 wt% CA/82 wt% NMP was chosen as a basic control, 18 wt% CA/1 wt% polyGMA/81wt% NMP and 18 wt% CA/1wt% polyGMA-CYS/81wt% NMP were prepared to demonstrate the polymer evolution, and finally 18 wt% CA/1wt% polyGMA-CYS-AgNP/81 wt% NMP and 18 wt% CA/2wt% polyGMA-CYS-AgNP/80 wt% NMP were prepared as the experimental groups. For convenience, these five dope solutions and corresponding membranes were named as shown in Table 1. 

The polymer(s) and solvent were mixed and sonicated for 12 h until clear solutions were obtained, then degassed for 60 min prior to casting. The viscosity of these dope solutions was measured using a rheometer (AG-G2, TA Instruments, New Castle, DE, USA) in the range of 0.1–10/s. 

**Figure 2:**
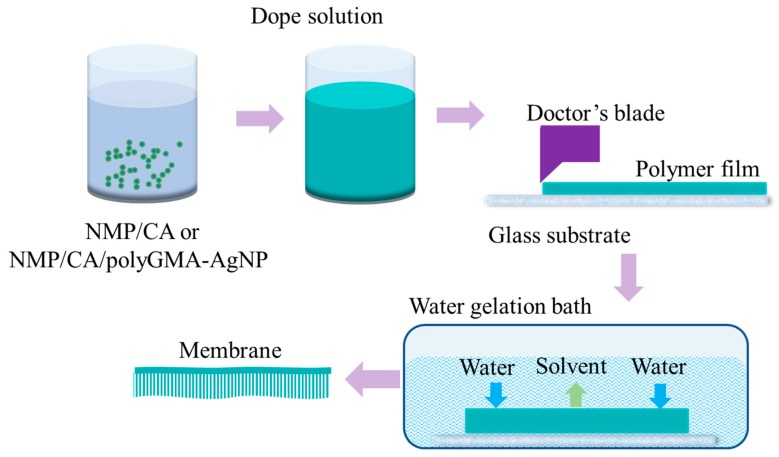
Nonsolvent-induced phase separation (NIPS) membrane fabrication method, in which membranes are cast using a doctor’s blade on a glass plate and immerse into deionized (DI) water as the non-solvent.

Nonsolvent-induced phase separation theory (NIPS) was the method used to cast membranes [33,34,35], as shown in Figure 2. A doctor’s casting blade with a thickness set as 200 μm was used to cast the thin film on a glass substrate at room temperature. The glass substrate was then immersed into the DI water gelation bath for phase inversion. Free standing membranes were obtained and stored in DI water for a week prior to using. Thickness of the membranes were measured at 100–120 μm using a deep throat digital thickness gage (547-520S, Mitutoyo Co., Kawasaki, Japan). 

The thickness difference before and after casting was analyzed in a previous study [35]. Water samples were taken from the post-casting gelation bath and the storing DI water to analyze if silver leached out during the casting and storage process. 

### 2.4. Membrane Characterization 

#### 2.4.1. Contact Angle/ Streaming Zeta Potential/ X-Ray Photoelectron Spectroscopy

The contact angle describes the hydrophilicity of the membrane, and it was analyzed using a Drop Shape Analyzer (Kruss, Hamburg, Germany). Each membrane sample was dried before testing and six readings were taken for accuracy with the average value calculated accordingly. 

Silver nanoparticles were immobilized on membrane surfaces, which influenced the membrane surface charge [36]. The surface charge of membranes was characterized by measuring the streaming zeta potential using an electrokinetic analyzer (Anton Paar SurPASS, Ashland, VA, USA). The adjustable gap cell was set as a gap of 90-110 μm using 0.01 M KCl as the electrolyte. 

Surface samples of five membranes were freeze-dried overnight to remove moisture and then analyzed using a K-Alpha X-ray photoelectron spectroscopy (XPS, Thermo Scientific, MA, USA). XPS provides information of elemental compositions based on the corresponding binding energies excited by X-ray beams. In this study, S_2p_, C_1s_, Ag_3d5/2_, Ag_3d3/2_, and O_1s_ core level peaks were observed verifying the immobilization of AgNP onto the membranes. 

#### 2.4.2. Scanning Electron Microscope/ Energy-Dispersive X-Ray Spectroscopy 

The morphology of polyGMA-CYS-AgNP polymer complex, along with the surface and cross-section morphologies of the membranes, were observed using a scanning electron microscope (SEM) (FEI Quanta Environmental SEM, Hillsboro, OR, USA) equipped with an energy-dispersive x-ray spectroscopy (EDX). SEM was used to image the polymer complex, membrane surfaces and cross-sections, while EDX was used to quantify the elemental composition on the membranes. 

The cross-sectional membrane samples were prepared by immersion into liquid nitrogen for 2 min and then carefully snapped into pieces. All the samples (polymer complex, surface membrane samples and cross-sectional samples) were kept frozen overnight and then placed in a freeze dryer (Labconco, Kansas City, MO, USA). All the samples were sputtered with palladium-gold (Quorum Emscope SC 400, Laughton, UK) for 4 min before imaging to minimize the accumulation of electrons on the membrane surfaces. 

### 2.5. Membrane Filtration Performance

Filtration experiments were conducted in a dead-end filtration cell (Amicon Stirred Cell 50 mL, Millipore Sigma Co., Burlington, MA, US) with an effective area of 13.4 cm^2^. The experiments were carried out at room temperature at a constant pressure of 0.4 MPa.

Precompaction of the membranes was performed using 20 mL DI water and followed by filtering 20 mL of 1 g/l BSA solution. BSA is a hydrophobic molecule with molecular weight of 66.5 kDa and Stokes radius of 3.48 nm [33,37]. The time required for filtering every 2 mL of DI water and BSA solution was recorded. 

### 2.6. Leaching Studies 

Leaching studies were carried out to investigate the strength of attachment of AgNPs to the membranes [24]. The membranes made from the dope solutions of M2 and M3 were investigated using both crossflow and a dead-end filtration studies. 

#### 2.6.1. Crossflow Filtration Studies

**Figure 3:**
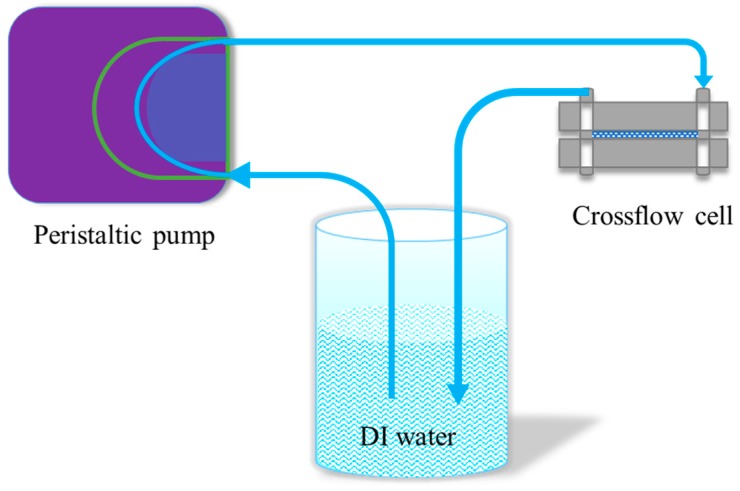
Crossflow leaching under full recycle experimental set-up.

Crossflow filtration was performed using a crossflow cell (Sterlitech CF042A, Sterlitech Co., Kent, WA, USA), as shown in Figure 3, with effective membrane sizes of 40 mm × 76 mm. Two pieces of plastic grid spacers were placed inside the flow cell to create turbulence. One L of DI water was filtered through the membrane using a peristaltic pump (Manostat Vera, Cole-Parmer Instrument Co., Vernon Hills, IL, USA) at a flowrate of 70–80 mL/min. The water passed through the membranes and then went into a recycling reservoir with continuous stirring by a magnetic stirring bar, and the water was then pumped back to the crossflow filtration station to pass through the membranes repeatedly. This formed a closed system. Five-ml water samples were taken every day for seven days, and two drops of 1 mol/L HCl were added into the samples to prevent potential bacterial degradation of Ag. All the water samples were stored in a dark walk-in cold room (at 4 °C). The concentration of silver leached from the membranes was determined using a Vista-Pro Ion Coupled Plasma Optical Emission Spectroscopy (ICP-OES) (Varian Inc., Palo Alto, CA, USA) equipped with argon plasma and a CCD detector. M2 and M3 were tested separately, and in triplicate for reproducibility. 

#### 2.6.2. Dead-End Filtration Studies 

Leaching studies were also performed using a dead-end filtration cell (Amicon Stirred Cell 50 mL, Millipore Sigma Co., Burlington, MA, USA). Circular pieces of membrane sample of 13.4 cm^2^ were cut from M2 and M3 and assembled in the filtration cell. DI water was filtered through the membranes. Five-ml samples were collected after 100, 250, 500 and 1000 mL of DI water past the membranes, and then the concentration of silver was determined using ICP-OES. Experiments were performed in triplicate. 

### 2.7. Antimicrobial Performance of Membranes

In this study, the ability to develop a visible bacterial lawn was used to determine the antimicrobial performance of the membranes embedded with AgNPs [38]. As previously stated, *Serratia marcescens* was chosen because it can produce red pigments under appropriate incubation conditions, and is not inhibited by low oxygen concentrations, which can potentially provide a protocol to visually determine the antimicrobial performance of polymeric membranes [26]. 

#### 2.7.1. Bacterial and Media Preparation

*Serratia marcescens* subsp. *marcescens* Bizio (ATCC 13880) was selected as a model bacterium to evaluate the inhibitory effect of different membranes. A pure culture of *S. marcescens* was incubated overnight at 27 °C in a Bacto^TM^ tryptic soy broth (TSB) to provide a sufficient bacterial population to form a bacterial lawn [38]. Fresh bacterial stock was prepared 24 h prior to each test and the number of bacteria was counted after serial dilution and spreadplating onto agar-containing petri dishes as 6.5 × 10^7^ CFU/mL. 

In this study, 1 L agar was made by adding 8 g of Difco^TM^ nutrient broth, 7 g of agar powder, and 5 g of 99.5% sodium chloride to 1 l of purified DI water and then autoclaved [38].

#### 2.7.2. Plate Preparation

Agar plates were prepared by transferring 10 mL of the autoclaved agar solution aseptically into each petri dish inside of a laminar flow hood. One agar plate was processed along sample plates as a negative media control to verify the sterility of the agar and plate preparation. All membranes were sterilized by immersing in ethyl alcohol and rinsing three times with autoclaved distilled water at room temperature. For each type of membrane, one negative control was prepared by gently laying a round sterilized membrane (13.4 cm^2^) onto an agar plate and incubating to verify the membrane sterilization procedure. One positive control agar plate was prepared by adding 2 mL of prepared bacterial stock of *Serratia marcescens* to 10 mL of liquid agar and pouring it into a petri dish to demonstrate the full growth of bacteria on the developed agar. All of the control groups were incubated at 27 °C for 24 h [26].

Membrane sample test plates were prepared by adding 2 mL of prepared bacterial stock into 10 mL of liquid agar solution and solidifying in room temperature. Then, a round sterilized membrane sample was gently laid on top of each agar plate. For each type of membrane, three replicate Petri dishes were used for reproducibility. All the test plates were incubated at 27 °C for 24 h. 

After the incubation, all the membranes on the test plates were gently removed. Test plates and control plates were observed on a colony counter, and the transparency of the bacterial lawn on different plates was compared visually to evaluate the antimicrobial performance of the membranes. 

#### 2.7.3. Fourier Transform Infrared Spectroscopy (FTIR) and RGB Color Analysis

The membranes on all the agar plates were gently removed after 24 h of incubation and then dried overnight in a desiccator to prepare them for attenuated total reflectance Fourier transform infrared spectroscopy analysis (ATR-FTIR, Nicolet^TM^ iS50 FT-IR, Thermo Scientific, Waltham, MA, USA). FTIR was used to qualitatively characterize the chemical structures of the five membranes before and after microbial growth. The contact diamond was cleaned using isopropanol prior to measuring each sample. 

The remaining agar plates, including negative control, positive control and the experimental plates, were compared. Photos were taken under the same light conditions. The colors of these plates were encoded into the RGB code system [39,40,41]. For each plate, nine points were chosen to calculate the average value. The details and one example can be found in the Supplementary Materials. Then the color difference (CD) of different plates were calculated by the following equation [42,43]:

(1) $$CD=\sqrt{{\left(R_{i}-R_{0}\right)}^{2}+{\left(G_{i}-G_{0}\right)}^{2}+{\left(B_{i}-B_{0}\right)}^{2}}$$

where 0 represents the negative control group and *i* represents the positive control group or the experimental group; and *R*, *G* and *B* represent the colors red, green and blue, respectively. 

The negative control was chosen as the reference object. The CD value between the positive control and the negative control was considered as 1, and then CD values between other groups and the negative control were normalized accordingly to digitalize the antimicrobial performance of different membranes. 

## 3. Results and Discussion 

### 3.1. Morphology and EDX Analysis of Polymer Complex

**Figure 4:**
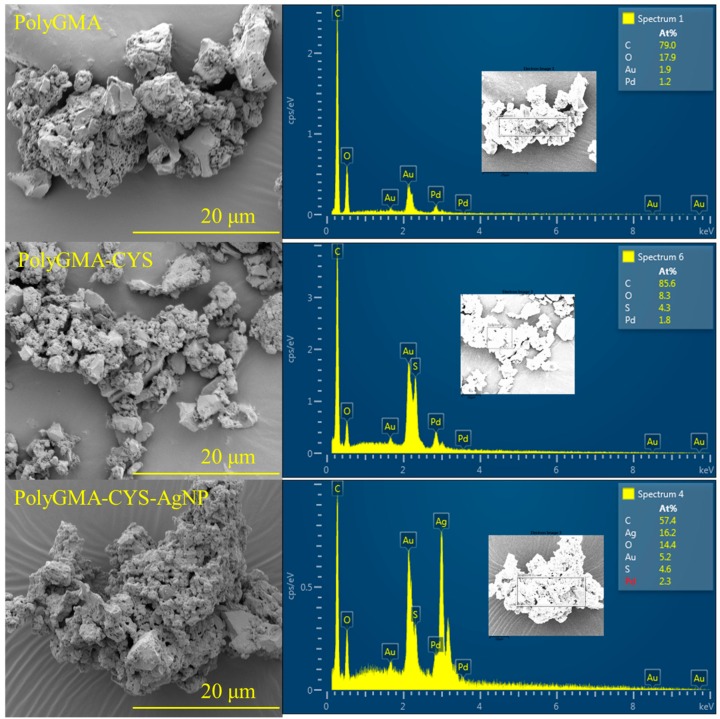
SEM Morphology images and EDX analysis of the polymer complex: from left to right and top to bottom: polyGMA showing major elementals components C and O; polyGMA-CYS showing appearance of S; and polyGMA-CYS-AgNPs showing the additional appearance of Ag.

Figure 4 shows SEM and EDX images of the evolution from polyGMA to polyGMA-CYS and then polyGMA-CYS-AgNP. As shown in Figure 1, monoGMA was polymerized into polyGMA, and then the primary amine groups of CYS were reacted with the epoxide groups of polyGMA to form polyGMA-CYS. Lastly AgNPs were added to the polymer complex via affinity to CYS thiol groups, which resulted in the polyGMA-CYS-AgNP polymer complex. The casein-coated AgNPs were characterized via TEM in a previous study, and the particle size distribution of the nanoparticles was reported to be 12.3 ± 1.9 nm [24], also agreeing with other literature studies [44,45].

SEM images show that the porous polymer complex was distributed within the range of 2–4 μm, and the size did not change significantly during the progress. The porous structure of the polymer complex provided a large surface–volume ratio, leading to more sites for the attachment reactions. The EDX results showed the presence of S and S/Ag, which qualitatively proved that CYS and AgNPs were successfully attached onto the polymers. The presence of Au and Pd was due to the coating prior to imaging. 

### 3.2. Membrane Characteristics

#### 3.2.1. Dope Solution Viscosity 

**Figure 5:**
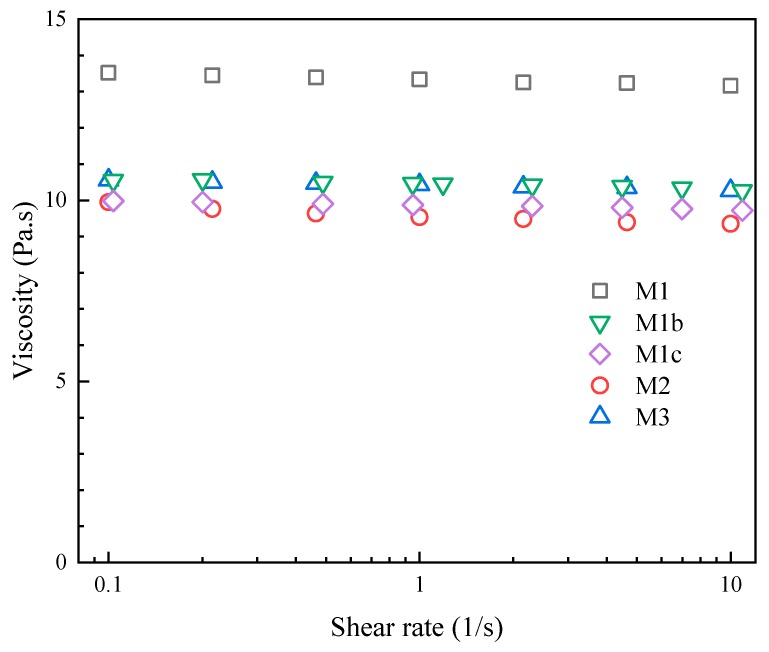
Viscosity of five dope solutions as a function of shear rate in the range of 0.1–10 /s.

For laboratory-scale casting, a doctor’s blade is often used to cast membranes [29,33,34,35,46]; therefore, the dope solution should be a Newtonian fluid because the viscosity does not change over the shear rate [29,34,35]. Investigating the viscosity behavior over the shear rate of the dope solution is important because it is the fundamental information required if a larger scale casting is considered. Figure 5 shows that the average viscosity value of the M1 dope solution was 13.4 Pa.s in the shear rate range of 0.1–10 /s. Since the viscosity did not change when the shear rate changed, the M1 dope solution was considered to be a Newtonian fluid. From M1 to M1b, adding polyGMA reduced the viscosity of the dope solution from 13.4 to 10.5 Pa.s. The viscosity of M1b, M1c, M2 and M3 dope solutions were all approximately 10 Pa.s, and they did not change significantly over the shear rate at 0.1–10 /s. The Newtonian fluid behavior of the produced dope solutions made them suitable for lab-scale casting using a doctor’s blade [35]. 

#### 3.2.2. Membrane Morphology

**Figure 6:**
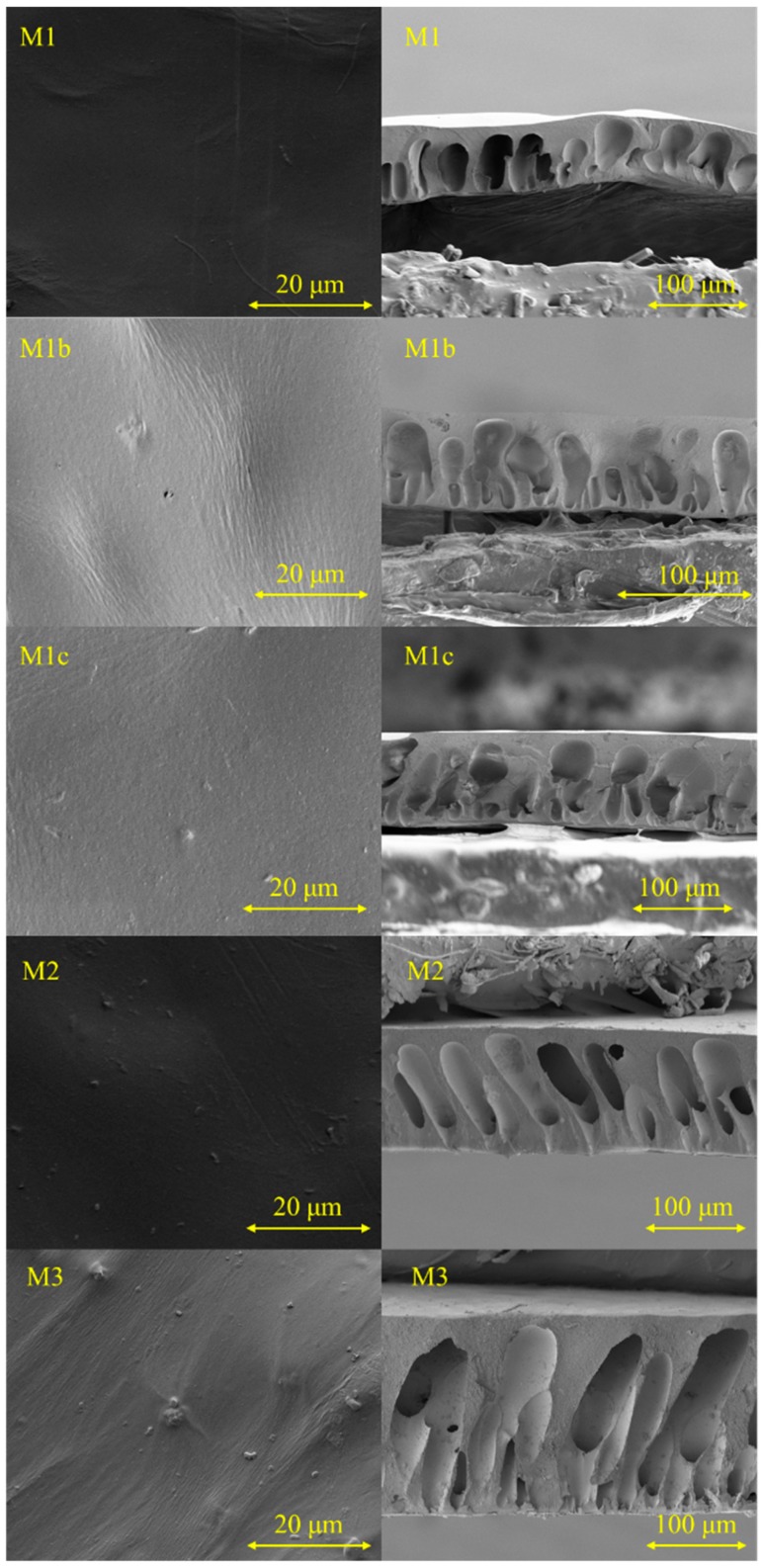
Surface and cross-section morphology images of membranes: from top to bottom: M1 represents the pristine cellulose acetate (CA) membranes, M1b is CA/polyGMA membranes, M1c is CA/polyGMA-CYS, M2 is CA/1% polyGMA-CYS-AgNP and M3 is CA/2% polyGMA-CYS-AgNP.

Figure 6 shows the surface and cross-sectional images of the membranes. All the five membranes did not present any defects on the surface. However, there were some solid particles on the surface of M2 and M3, which might have been due to some impurities or particles on the membrane surface. Cross-sectional images showed that all membranes displayed finger-like structures, which is the typical cross-section morphology for CA/NMP ultrafiltration membranes without using additives [24,47]. From M1 to M1b, M1c and M3, the microvoids changed from the regular finger-like structures to the irregular finger-like channels along with some small channels appearing on the 2% polymer complex membranes. M2 was formed by adding 1% polymer complex in the dope solution; therefore, the channels presented a morphology with similarities between M1 and M3; that is, a regular finger-like structure with the presence of less small channels. 

#### 3.2.3. Contact Angle

**Figure 7:**
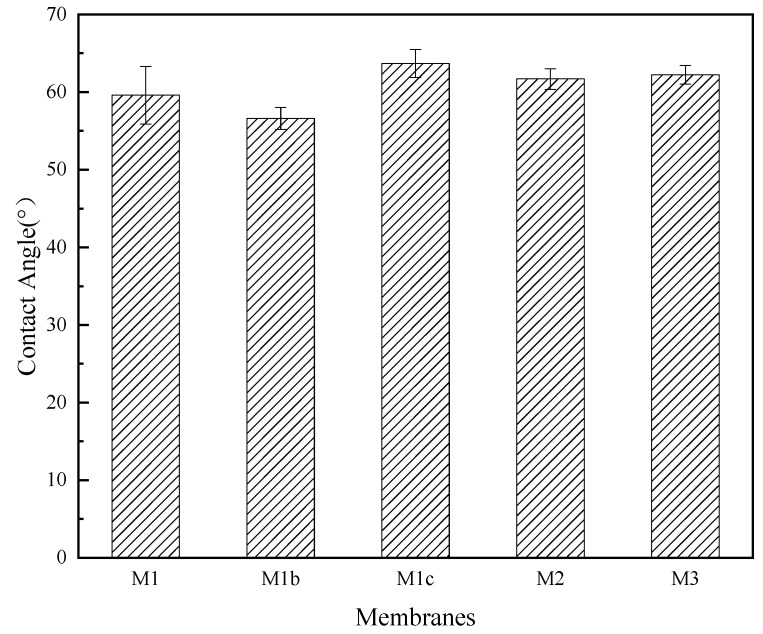
Contact Angle of the five membranes fabricated showing no significant differences.

Figure 7 presents the evolution of the contact angle of membranes as the membranes were modified, describing their hydrophilicity. The contact angle of the baseline CA membrane (M1) was measured at 59.6 ± 3.7°. The contact angles of modified membranes were not significantly different from M1, which agreed with the reported work [24]. Hydrophilicity of membranes affects the water permeability [48,49]; therefore, the addition of the polymer complex and of AgNPs were not expected to negatively impact permeability. 

#### 3.2.4. X-ray photoelectron spectroscopy

**Figure 8:**
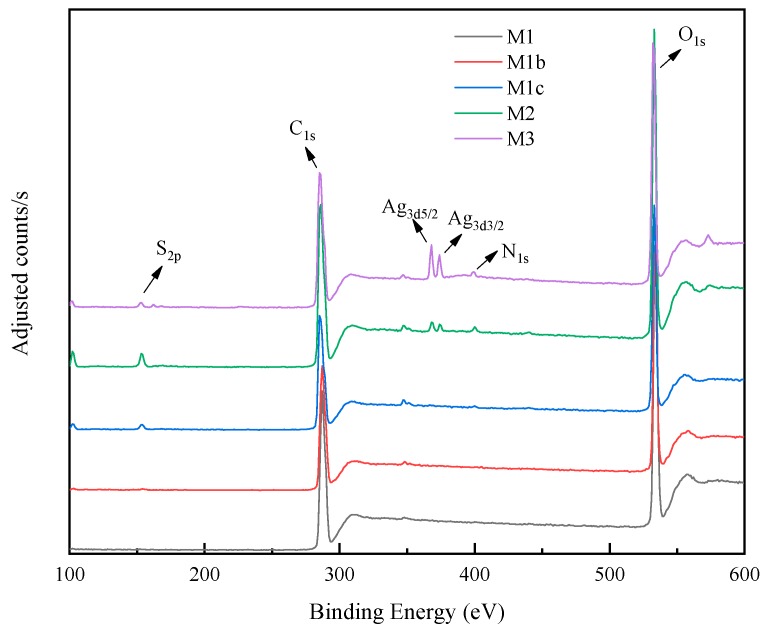
X-ray photoelectron spectroscopy (XPS) spectra of the membranes: M1 (bottom curve) was the baseline CA membranes showing the presence of only carbon (C) and oxygen (O); M1b (second from the bottom) showing only C and O elements consistent with the structure of CA polyGMA; M1c (third from the bottom, CA with polyGMA-CYS) showing the addition of S; lastly, M2 and M3 (top two curves with the addition of AgNPs) indicating the additional presence of silver.

XPS was used to identify the elements on the membrane surfaces, as shown in Figure 8. M1 and M1b presented similar spectra with major C_1s_ and O_1s_ peaks, which was expected, since polyGMA has the same elements as CA. M1c presented the S_2p_ peak associated with the addition of thiol groups. The spectrum of M2 and M3 showed the presence of S_2p_ and two Ag peaks (Ag_3d5/2_ and Ag_3d3/2_), supporting that the reactions which occurred since the targeted elements were incorporated on the membrane surface. 

#### 3.2.5. Streaming Zeta Potential

**Figure 9:**
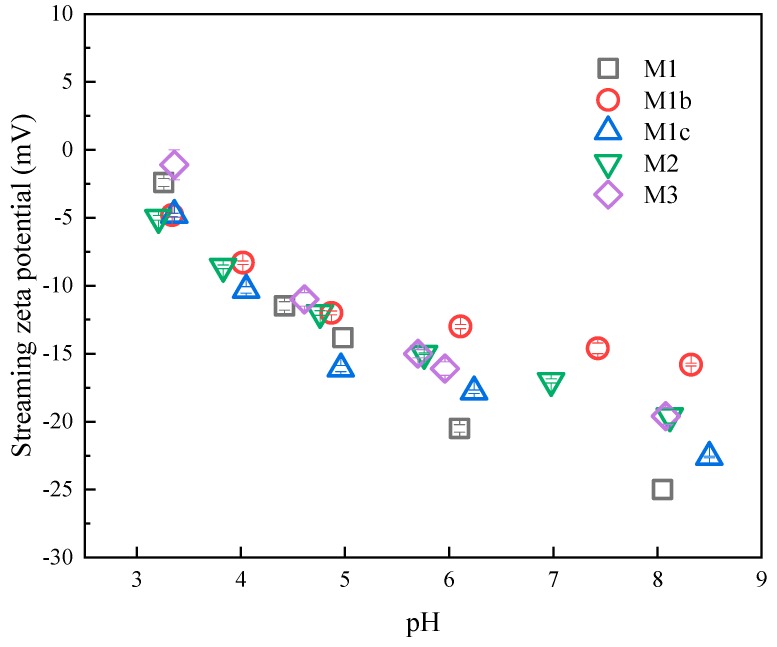
Streaming zeta potential spectrum as a function of pH showing the charge evolution of the membrane modifications.

Streaming zeta potential is used to demonstrate the surface charge of the solid materials in an aqueous solution [36,50]. It is an important parameter to understand surface chemistry and the interactions between the surface and the surrounding environment [36]. Figure 9 shows the evolution of the zeta potential on the membrane surfaces as the polymer complex and the nanoparticles were added to the base CA membranes. Zeta potential values were observed not to be significantly different when the aqueous environment was acid (<5). As pH increase to 6, the range broadened. The baseline CA membrane (M1) was negatively charged because it was made of cellulose acetate, of which the negative-charged acetyl group is the major functional group [51]. Then the pH value increased above 6, and the results showed that M1b presented a less negative surface charge as compared to M1, which might have been due to the presence of epoxide groups on polyGMA. Epoxide groups are not as negative as acetyl groups [52,53]; therefore, the membranes were less negatively charged. With the addition of CYS, the membrane surface became more negative because the thiol groups were reacted with the epoxide groups. In this reaction, the epoxide rings broke, and thiol groups were added, which added a negative electron donor, sulfur. This is the likely reason why M1c was more negative than M1b. The attachment of AgNP consumed the negative charges added by sulfur, resulting in the phenomenon that the M2 and M3 were not as negative as M1c. The difference of surface charge on membranes was another evidence that supported the surface modification reactions. 

### 3.3. Leaching Study 

#### 3.3.1. Crossflow Leaching

**Figure 10:**
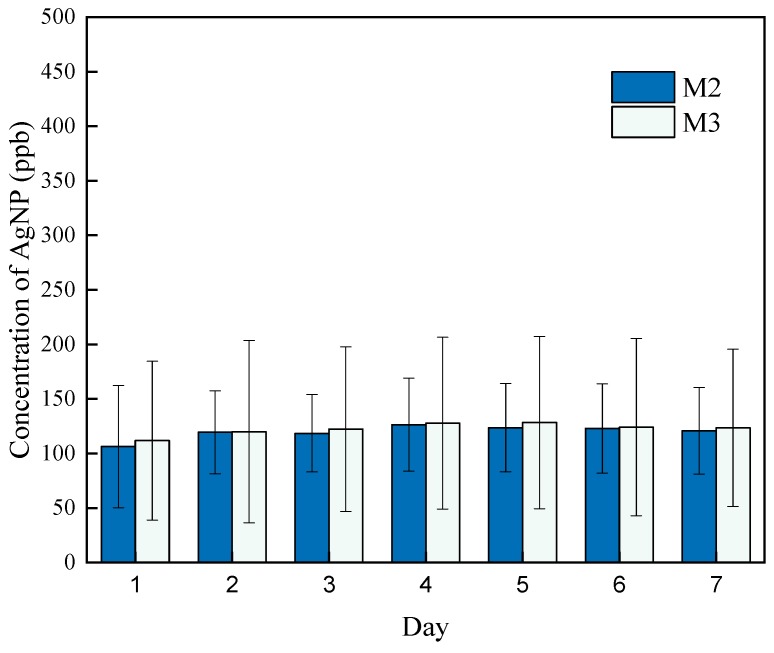
Crossflow leaching study of the membranes showing the concentration of silver that leached from M2 and M3 membranes between days 1 and 7.

DI water was filtered in crossflow studies under full recycle for 7 days to determine the leachability of AgNPs incorporated on the membranes. 5 mL aqueous samples were taken every day and the concentration of silver was analyzed using ICP-OES. Figure 10 shows that the leaching concentration for both M2 and M3 reached a peak value on the first day of testing and remained approximately constant through the last day of test. This was likely due to some unbound AgNPs leaching into the DI water, and due to the full-recycle setup, the value remained constant. 

A mass balance was then calculated. For a piece of membranes M2 and M3 of 30.4 cm^2^ and thickness of 150 μm, the mass of polymer used was approximately 0.3 g. Based on the recipe described in the Methods Section, the original amount of silver element contained in this size of membrane would be 4.6 mg in M2 and 8.7 mg in M3, respectively. In the leaching study, 1 L of DI water was used, and based on Figure 10, the amount of silver leached out from M2 and M3 would be 0.16 ± 0.06 mg/l and 0.19 ± 0.04 mg/l, respectively. Approximately, the silver remaining on M2 would be 4.44 mg and on M3, 8.51 mg. Based on the calculation, in the 7-day crossflow study in a full recycle mode, 97% of Ag would remain on M2 and 98% would remain on M3. Therefore, the leaching was minimized via the chemical attachment method. 

#### 3.3.2. Dead-End Leaching

**Figure 11:**
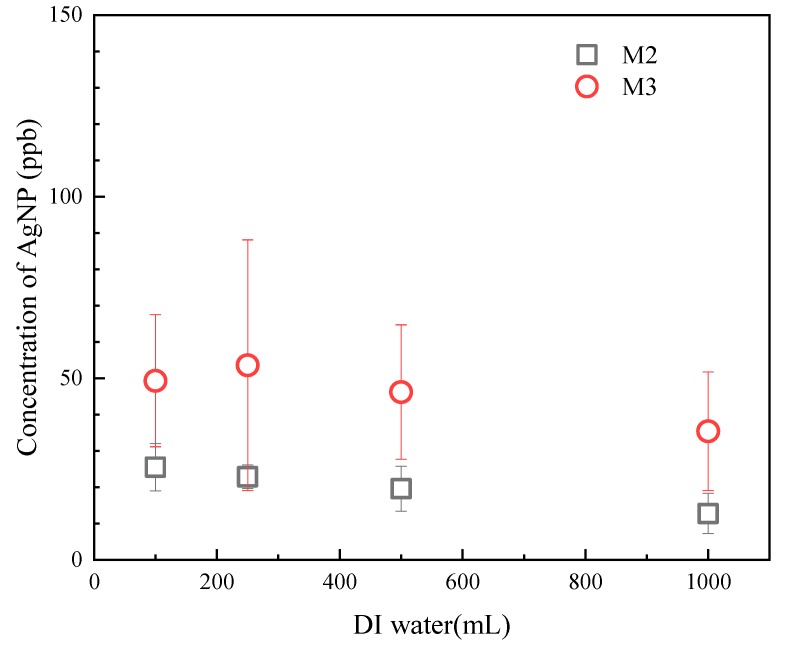
Dead-end leaching study of the membranes: concentrations of silver in the permeate as a function of the volume of DI water filtered through the membranes.

Figure 11 shows the change in silver concentrations when 1 L of DI water was filtered using a dead-end filtration cell. For M2, the silver concentrations in the permeate after filtering 100, 250, 500 and 1000 mL DI water were 25.5 ± 6.5, 22.9 ± 3.3, 19.6 ± 6.2 and 12.8 ± 5.6 μg/l, respectively. For M3, the silver concentrations in the permeate after the filtration of 100, 250, 500 and 1000 mL DI water were 49.3 ± 18.2, 53.6 ± 34.5, 46.2 ± 18.5 and 35.4 ± 16.3 μg/l, respectively. A declining trend of silver concentrations was observed as more water filtered through the membranes, which agreed with the results of the crossflow leaching supporting that this was the flushing of any unbound AgNPs. Furthermore, the concentration of silver that leached from M3 was higher than M2, as expected since M3 was loaded with more AgNPs. 

### 3.4. Membrane Filtration Performance 

**Figure 12:**
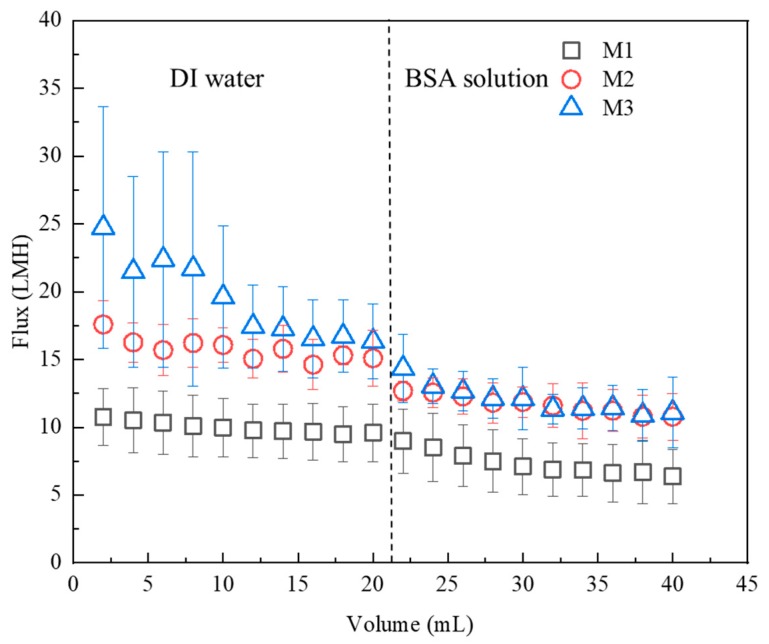
Filtration performance of the membranes as measured by flux decline of the baseline (M1) and two membranes with AgNPs (M2 and M3) as a function of the volume of BSA filtered through the membranes. The pressure remained constant at 0.4 MPa.

The leaching studies showed that the AgNPs were strongly incorporated onto the membranes with minimal loss, so filtration studies were then performed to evaluate the permeability of membranes containing the AgNPs, namely M2 and M3, as compared to the baseline CA membrane, M1. Figure 12 shows that M2 and M3 had higher flux values as compared to M1 during both DI water precompaction and BSA filtration. Initially, M2 and M3 displayed water flux values of 18 ± 2 L/m^2^ hr (LMH) and 25 ± 9 LMH, respectively, and both were higher than that of M1 (11 ± 2 LMH). After precompaction, M2 and M3 displayed water flux values 15 ± 2 LMH and 16 ± 3 LMH, respectively, and while both were higher than that of M1 (10 ± 2 LMH), they were not significantly different. During the precompaction process, the flux of M3 changed the most while that of M1 changed the least. Given that the hydrophilicity values of all membranes were in the same range as shown in Figure 7, the reason might be that the cross-section structures of these membranes were different (Figure 6). Specifically, AgNPs behaved as pore formers on the membranes [24,54,55] to build more numerous small water channels in the cross-sections of membranes. Therefore, with the addition of AgNPs, the water flux of membranes increased. 

The flux declined during BSA filtration for all membranes, as expected since the membranes likely fouled due to the BSA accumulation on the membrane surfaces. As observed after precompaction, the membranes with the lowest flux values were the baseline CA membranes (M1) with 6 ± 2 LMH, and the two membranes modified with AgNPs displayed similar flux values of 11 ± 2 LMH for M2 and 11 ± 3 LMH for M3. 

### 3.5. Antimicrobial Performance

**Figure 13:**
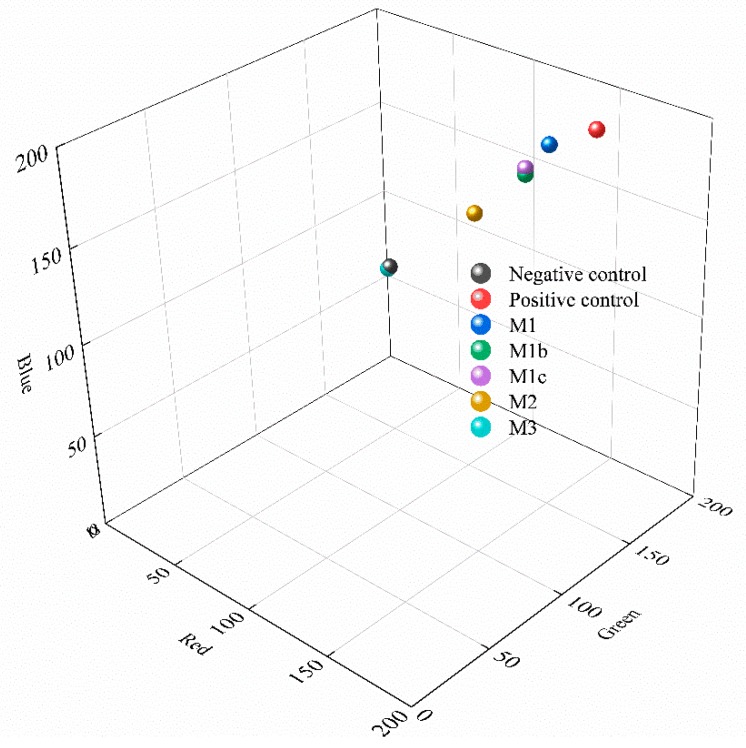
Quantitative color differences after 24 h of bacterial growth study, where the x axis is red, y axis is green and z axis is blue: negative and positive control were at the two ends, the pristine membrane M1 was close to the positive control, and membranes incorporated with AgNPs (M2 and M3) showed inhibition of bacterial growth.

Colors of the agar plates with bacteria lawns (images shown in Supplementary Materials) were coded into the 256 bits RGB model, where red represents the X axis, green represents the Y axis and blue represents the Z axis. As shown in Figure 13, the color of the positive control represents full growth and the color of negative control represents zero growth, and all membrane growth experimental results were between these two ends. As expected, the baseline membrane M1 was close to the positive control, indicating near biofouling formation, while the colors of transitional membranes M1b and M1c showed slightly less microbial growth as compared to that of M1. With 1% polyGMA-CYS-AgNP (the M2 membranes), significant inhibition of bacterial growth was observed. Lastly, M3 (2% polyGMA-CYS-AgNP), the membrane with the highest concentration of AgNPs, was located close to the negative control group, which means that this polymer complex completely inhibited the growth of the bacterial lawn on agar plates.

The color differences (CD) were calculated and are shown in Table 2. The positive control was set as 100% and the negative control was set at 0%. The normalized CD of M1 was 81%, M1b was 67% and M1c was 68%. These results showed that there was minimal inhibition of bacterial growth by these membranes on agar surfaces. For M2, with 1% polyGMA-CYS-AgNP, the normalized CD was measured as 41%, showing a significant suppression of bacterial growth. With 2% polyGMA-CYS-AgNP, the normalized CD of M3 was only 1%, close to the negative control, showing an almost complete suppression. 

The FTIR analysis on the surface of membranes after 24h of bacterial growth indicated the presence of nitro groups on M1, M1b and M1c, and their absence on M2 and M3. Nitro groups are fundamental functional groups to form proteins, DNAs and RNAs, which are the basic compositions of a biofilm [56]. Therefore, the results indicated that the bacterial reproduction and metabolism on the plates of M2 and M3 (membranes incorporated with AgNPs) were not as active as M1, M1b and M1c (membranes without AgNPs). The FTIR results further support that membranes developed here inhibited microbial growth. Detailed analysis is attached in the Supplementary Materials. 

## 4. Conclusions

In this study, casein-coated AgNPs were chemically attached on CA membranes to minimize the leaching of particles. AgNPs were attached through a procedure where the epoxide groups of polyGMA were open and attached with CYS, which was then followed by the addition of thiol groups (from CYS) attached with AgNPs. The polymer complexes were then added to the membrane dope solution to minimize the number of post-synthesis steps. 

The addition of polyGMA-CYS-AgNPs changed the cross-sectional structures by producing irregular structures and generating more small channels. XPS results verified the presence of sulfur and silver on the membranes cast with CYS and AgNPs, respectively. Dead-end and crossflow leaching studies showed little leaching of AgNPs. During BSA filtration studies, membranes containing AgNPs (M2 and M3) showed higher flux values as compared to the baseline CA membranes (M1), likely due to the presence of the NPs acting as pore formers.

Lastly, membranes incorporated with AgNPs showed significant suppression of *Serratia marcescens*, as compared to membranes without AgNPs. M2 displayed 41% of the full growth, while M3 showed only 1% of full bacterial growth. The results indicate that the membranes incorporated using AgNPs effectively suppressed the growth of *S. marcescens*. The results also demonstrated an improved method for visualizing bacterial growth, or lack of growth, using an organism whose growth is not suppressed by a lack of oxygen, and can provide a pigmentation signal to measure.

## Figures and Tables

**Table 1:** Recipe of dope solutions.

Label (wt%).	M1	M1b	M1c	M2	M3
CA	18	18	18	18	18
NMP	82	81	81	81	80
PolyGMA		1			
PolyGMA-CYS			1		
PolyGMA-CYS-AgNP				1	2

**Table 2:** Red, green, blue (RGB) values of colors of the petri dishes after 24 h of bacterial growth.

	R	G	B	CD	Normalized CD
Negative control	91.5	108.3	127.3	0.0	0%
Positive control	156.3	175.5	190.5	112.8	100%
M1	139.3	163.5	182.5	91.6	81%
M1b	133.3	154.3	169.5	75.1	67%
M1c	133.0	154.4	172.3	76.7	68%
M2	117.5	137.6	151.4	46.0	41%
M3	90.9	107.9	126.3	1.1	1%

## Supplementary Materials

The following are available online at https://www.mdpi.com/2073-4360/11/12/2057/s1, Figure S1: Digitalization of the colors: (a) open the photo in Paint 3D; (b) randomly choose 9 points on the agar plate and extract the color using eyedropper; (c) read the RGB values in the current color. Figure S2: FTIR spectrum on the membranes after incubation of 24 h: from left to right and top to bottom: M1 is the pristine membrane, M1b is CA/polyGMA membrane, M1c is CA/polyGMA-CYS membrane, M2 is CA/1% polyGMA-CYS-AgNP membrane, M3 is CA/2% polyGMA-CYS-AgNP membrane. M1, M1b and M1c showed a peak at 1540 cm-1 after incubation while M2 and M3 did not show the same peak.
