# Supplementary material for: Thiol-Affinity Immobilization of Casein-Coated Silver Nanoparticles on Polymeric Membranes for Biofouling Control
Source: Polymers (Basel). 2019 Dec 11;11(12):2057. doi: 10.3390/polym11122057 (PMC6961038; doi:10.3390/polym11122057)
Supplement: Supplementary file 1 [file polymers-11-02057-s001.pdf]

Supplementary Materials

Article

Thiol-Affinity Immobilization of Casein-Coated Silver Nanoparticles on Polymeric Membranes for Biofouling Control

Xiaobo Dong [1], Halle D. Shannon [1], Atena Amirsoleimani [2], Gail M. Brion [2] and Isabel C. Escobar [1,*]

[1] Department of Chemical and Materials Engineering, University of Kentucky, Lexington 40503, KY, USA; xiaobo.dong@uky.edu (X.D.); halledanielle218@uky.edu (H.D.S.)
[2] Department of Civil Engineering, University of Kentucky, Lexington 40503, KY, USA; aam227@uky.edu (A.A.); gail.brion@uky.edu (G.M.B.)
* Correspondence: Isabel.Escobar@uky.edu; Tel.: +1-859-257-7990

I. Photos of the agar plates

Agar plates were prepared in prior to the experiments.

Negative control: three agar plates without introducing any bacteria.

Positive control: *Serratia marcescens* (ATCC #13880) was introduced on the agar plates.

Experimental groups: Five membranes (M1, M1b, M1c, M2, and M3) were laid on the surface of the agar plates with *Serratia marcescens*.

All the plates were incubated at 27°C for 24h. After incubation, the membranes were carefully removed from the agar plates and photos were taken to demonstrate the growth of bacteria. All the materials used in this experiment were autoclaved and sterile. The photos are listed below:

The photo of negative control:

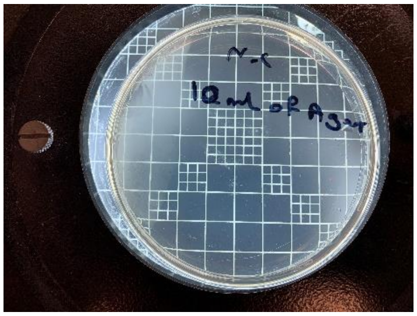

No bacteria grew on the agar plates after 24 h of incubation.

The photos of positive control:

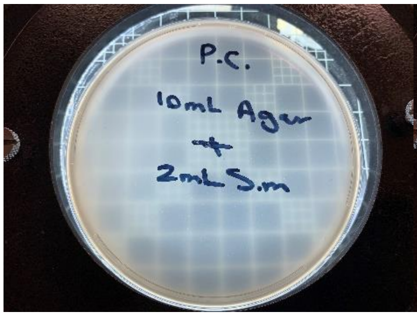

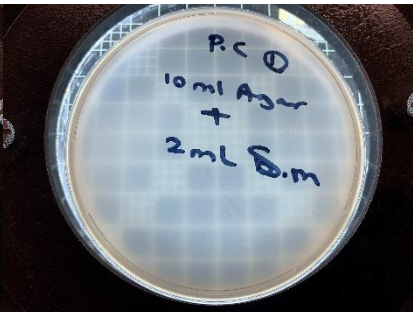

The positive control showed the full growth of *Serratia marcescens* after 24 h. A homogenous bacteria lawn was clearly observed on each agar plate. There were no obvious red pigments showed on the plates, but the agar plates showed a light pink color compared to the negative control. This color is set as the color of full growth.

The photos of membrane M1:

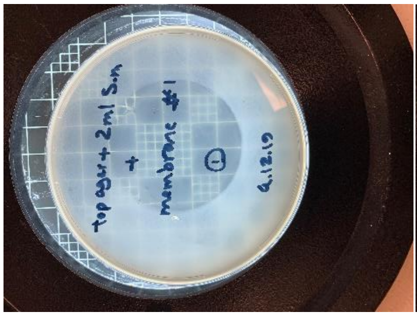

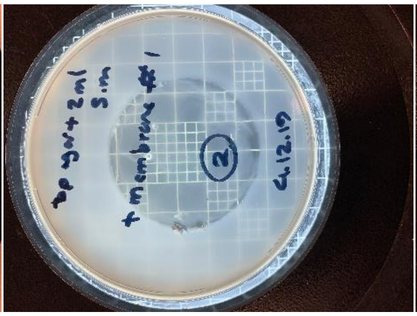

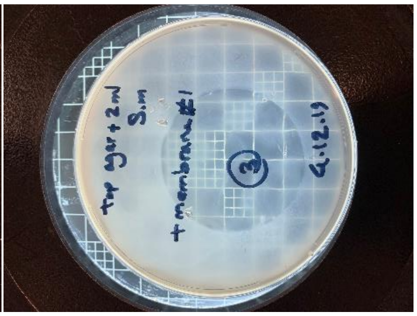

The photos of membrane M1b:

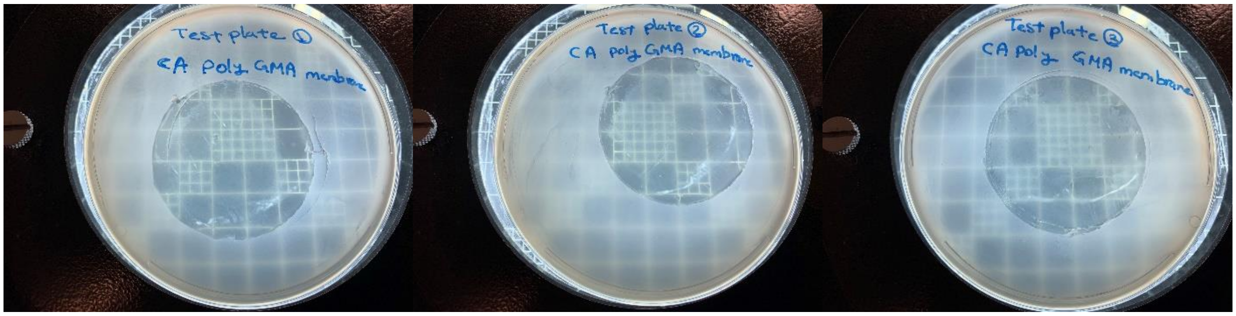

The photos of membrane M1c:

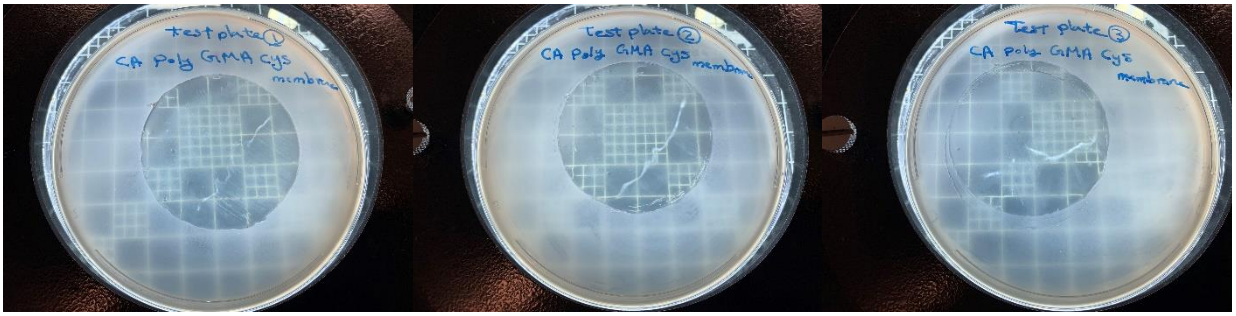

The photos of M1, M1b and M1c showed that after 24 h of incubation, the pure CA and the transitional membranes showed slight suppression of growth, but bacteria were still able to grow on the agar. The slight suppression is likely due to the shift in metabolism caused by the presence of membranes reducing the direct contact of agar with air, decreasing the availability of oxygen to serve as a terminal electron acceptor, therefore slowing the growth of bacteria and production of pigment.

The photos of membrane M2:

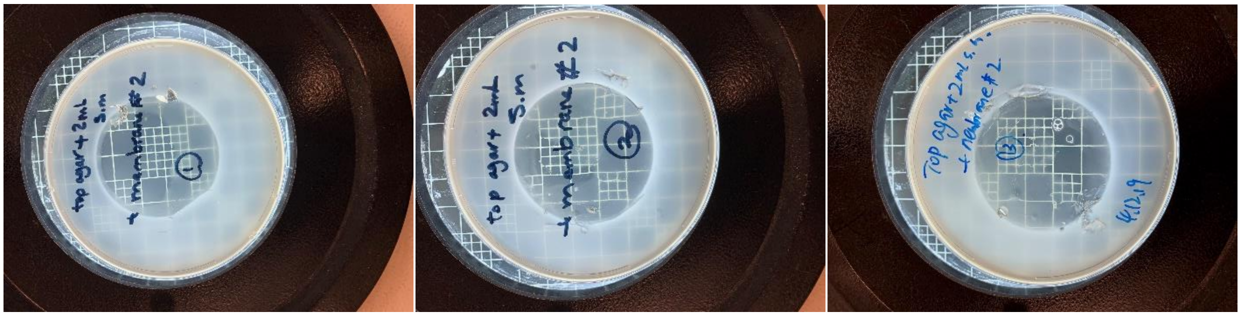

The photos of membranes M3:

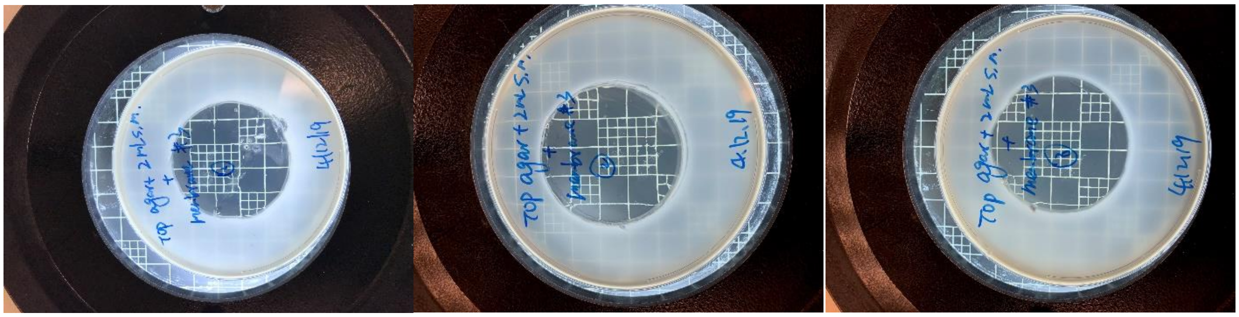

Photos of M2 and M3 showed an unambiguous inhibition of bacterial growth. M2 was observed to be more effective than M1, M1b and M1c, while M3 showed the best antimicrobial performance of all the membranes. The M3 plates showed a clearing spot visually indistinguishable from the negative control, thus, M3 completely suppressed the growth of Serratia marcescens.

The colors of the negative control, positive control and the experimental groups were coded using the 256 bits RGB model to quantify the antimicrobial performance of different membranes.

II. Digitalization of colors on agar plates

Digitalization of colors was performed using the software Paint 3D (Microsoft Corporation, Version 6.1907.18017.0). In this method, the positive control agar plate was chosen as a template and the detailed procedures are showed in Figure 1. The photo was opened in the Paint 3D as Figure 1 (a). Nine points of the test area were then randomly chosen, and for each point, the tool of eyedropper was used to extract the color of this point, as shown in Figure 1 (b). The corresponding red-green-blue (RGB) values were read in the current color, as shown in Figure 1 (c). After obtaining the RGB values of all these 9 points, the average and deviation was calculated to represent the color of the agar plate.

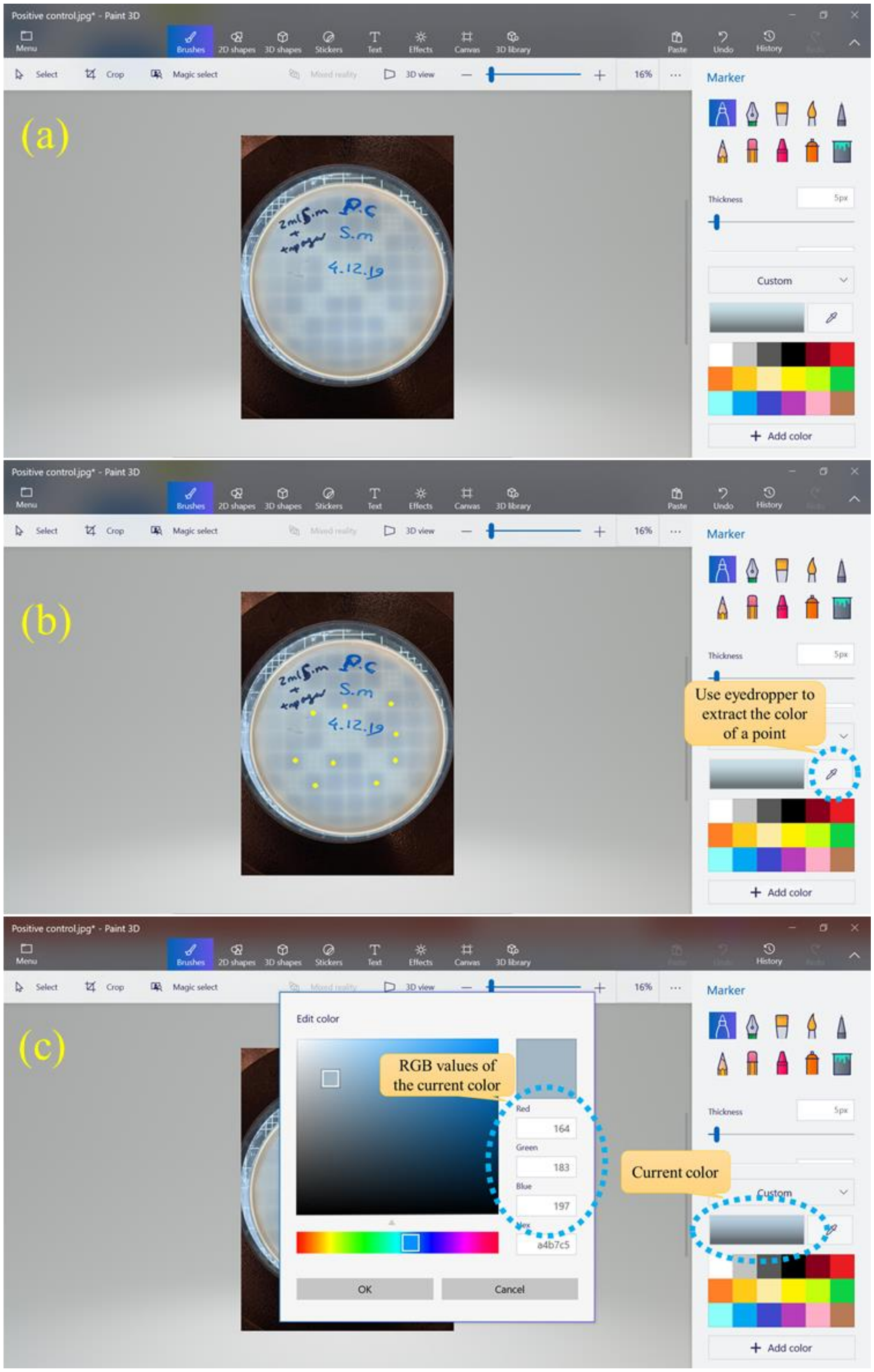

Figure 1 Digitalization of the colors: (**a**) open the photo in Paint 3D; (**b**) randomly choose 9 points on the agar plate and extract the color using eyedropper; (**c**) read the RGB values in the current color.

III. FTIR results

The five membranes after 24 h of bacterial incubation were collected and rinsed using DI water. For each membrane, the original and experimental membrane were dried in a freeze dryer overnight and then FTIR was performed to characterize the membrane surface.

Figure 2 shows that the major difference between the five membranes was the peak at ~1540 cm^{-1}, assigned to N-H bending coupled to C-N stretching. The peak represents the presence of nitro groups [1]. Nitro groups are fundamental functional groups to form proteins, DNAs and RNAs, which are the basic compositions of a biofilm [2]. After 24 h of bacterial growth, the FTIR spectrum of M1, M1b and M1c showed significant peaks at ~1540 cm^{-1}. This peak on the spectrum of M2 was negligible, and there was no peak at 1540 cm^{-1} on the M3 spectrum after 24h of bacterial growth. The peaks at 1540 cm^{-1} showed that the nitro groups on M2 and M3 were significantly smaller as compared to M1, M1b and M1c, indicating that the bacterial reproduction and metabolism on the plates of M2 and M3 (membranes incorporated with AgNPs) were not as active as M1, M1b and M1c (membranes without AgNPs). Therefore, coupled with the analysis of agar plate photos, FTIR results provided additional evidence that membranes incorporated with AgNPs showed the potential to inhibit microbial growth.

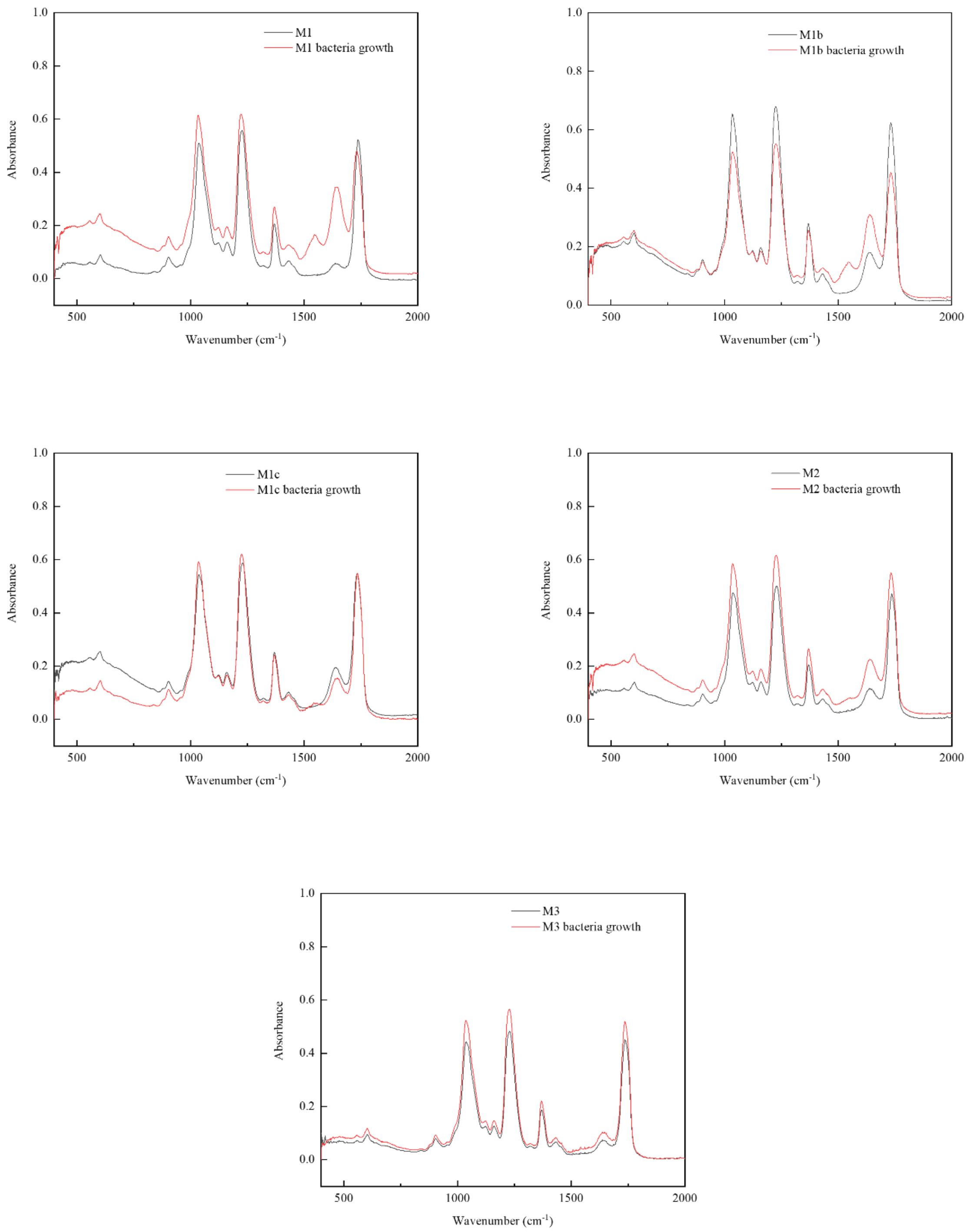

Figure 2 FTIR spectrum on the membranes after incubation of 24 h: from left to right and top to bottom: M1 is the pristine membrane, M1b is CA/polyGMA membrane, M1c is CA/polyGMA-CYS membrane, M2 is CA/1% polyGMA-CYS-AgNP membrane, M3 is CA/2% polyGMA-CYS-AgNP membrane. M1, M1b and M1c showed a peak at 1540 cm^{-1} after incubation while M2 and M3 did not show the same peak.

References:

1. Zhang, J.; Li, B.; Wang, Q.; Wei, X.; Feng, W.; Chen, Y.; Huang, P.; Wang, Z., Application of Fourier transform infrared spectroscopy with chemometrics on postmortem interval estimation based on pericardial fluids. *Scientific Reports* **2017,** *7* (1), 18013.

2. López, D.; Vlamakis, H.; Kolter, R., Biofilms. *Cold Spring Harb Perspect Biol* **2010,** *2* (7), a000398-a000398.
